# Supplementary material for: Granger Causality Analysis of Transient Calcium Dynamics in the Honey Bee Antennal Lobe Network
Source: Insects. 2023 Jun 9;14(6):539. doi: 10.3390/insects14060539 (PMC10299490; doi:10.3390/insects14060539)
Supplement: Supplementary file 1 [file insects-14-00539-s001.zip › insects-2398084-supplementary.pdf]

insects

MDPI

Supplementary Information

Supplementary Table S1. Statistical test on best-match-to-template tests. Wilcoxon signed rank test parameters: original *p*-values (black), FDR-adjusted *p*-values (red), and signed rank value *W* (blue). For each odor/bee combination, *n* = 15.

	Edge-centered						Node-centered	
	within			across			within	across
	ON	earlyOFF	OFF	ON	earlyOFF	OFF	ON	ON
1HEX	0.0001	0.013	0.72	0.0016	0.16	0.078	0.0002	0.07
	**0.0004**	**0.020**	0.72	**0.0010**	0.32	0.24	**0.0002**	0.11
	119	103	52	110	84	29	118	92
3HEX	0.0016	0.0022	0.30	0.45	0.45	0.0065	0.0001	0.0005
	**0.0032**	**0.0044**	0.59	0.54	0.54	0.04	**0.0002**	**0.0015**
	99	98	53	75	75	15	119	114
1NON	0.0034	0.0005	0.54	0.0056	0.07	0.32	0.0001	0.0001
	**0.0050**	**0.0015**	0.64	**0.012**	0.21	0.38	**0.0002**	**0.0007**
	109	78	42	105	92	42	105	119
ISOA	0.0001	0.0004	0.12	0.0056	0.016	0.45	0.0001	0.0056
	**0.0004**	**0.0015**	0.59	**0.012**	0.10	0.45	**0.0002**	**0.012**
	120	103	78	105	99	75	120	105
ACPH	0.018	0.28	0.52	0.32	0.32	0.16	0.0010	0.16
	**0.018**	0.33	0.64	0.47	0.47	0.32	**0.0010**	0.20
	101	71	42	42	42	84	101	84
BZDA	0.011	0.99	0.22	0.56	0.68	0.32	0.0001	0.71
	**0.013**	0.99	0.59	0.56	0.68	0.38	**0.0002**	0.71
	92	61	27	55	60	42	105	54

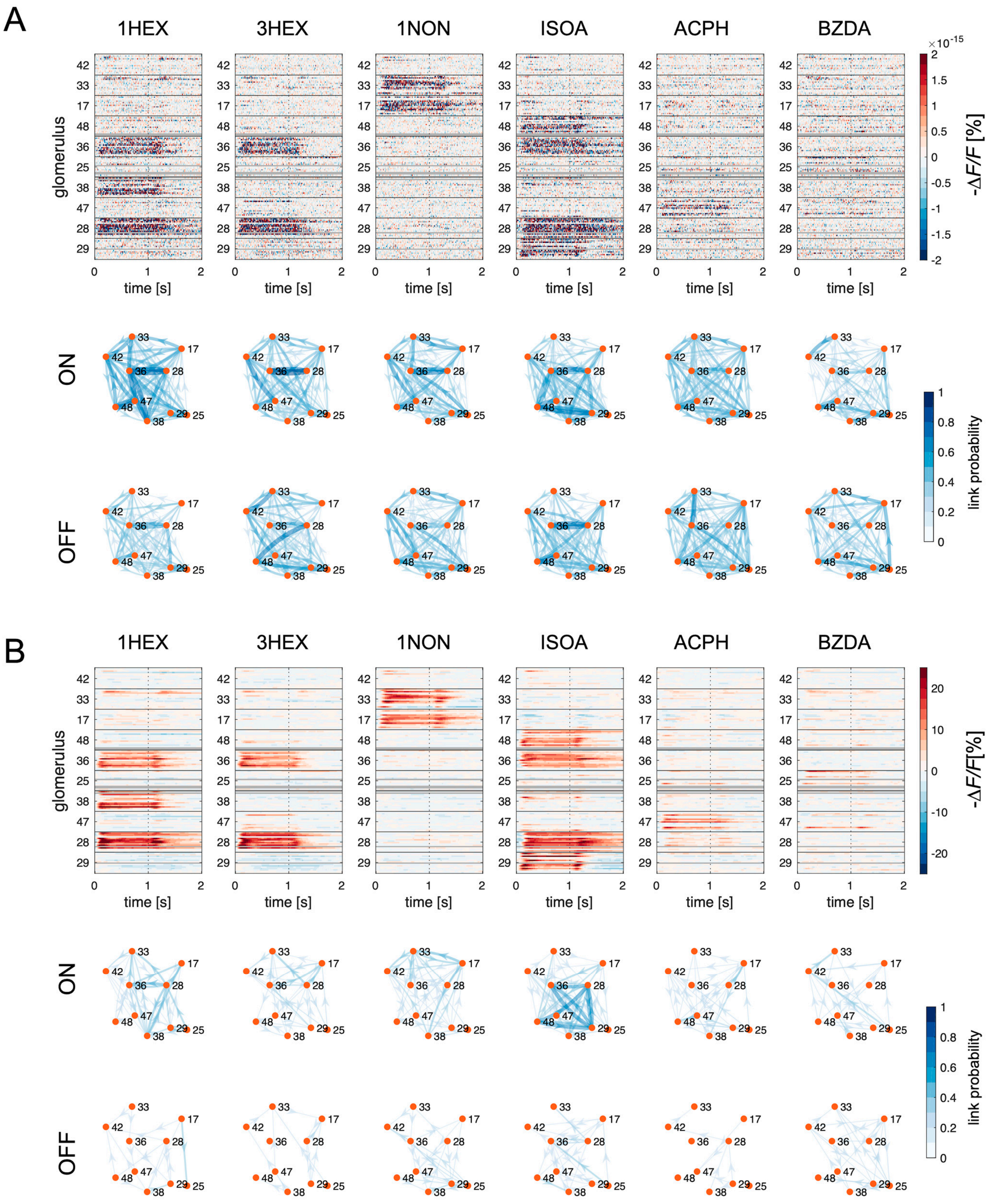

Supplementary Figure S1. Glomerular profiles and connectivity maps, computed from the fast (A) and slow (B) signal components. (A) Glomerular responses across bees and glomeruli. The relative fluorescence change is color-coded as a function of time, gray lines represent the unavailability of individual glomerular data in single bees. Olfactory stimulation is delivered in the 0-1s interval. The *y*-axis shows the response profiles of individual bees (n = 15) grouped according to the glomerulus ID number. Mean connectivity maps across all bees were calculated during stimulation (t = 0 to 1 s,

top row) and 5s after odor offset (*t*=6 to 7s, bottom row). (B) The same data are presented after filtering original calcium signals to preserve only the slow components. Abbreviations: 1-hexanol, 1HEX; 3-hexanol, 3HEX; 1-nonanol, 1NON; isoamyl acetate, ISOA; acetophenone, ACPH; benzaldehyde, BZAD.

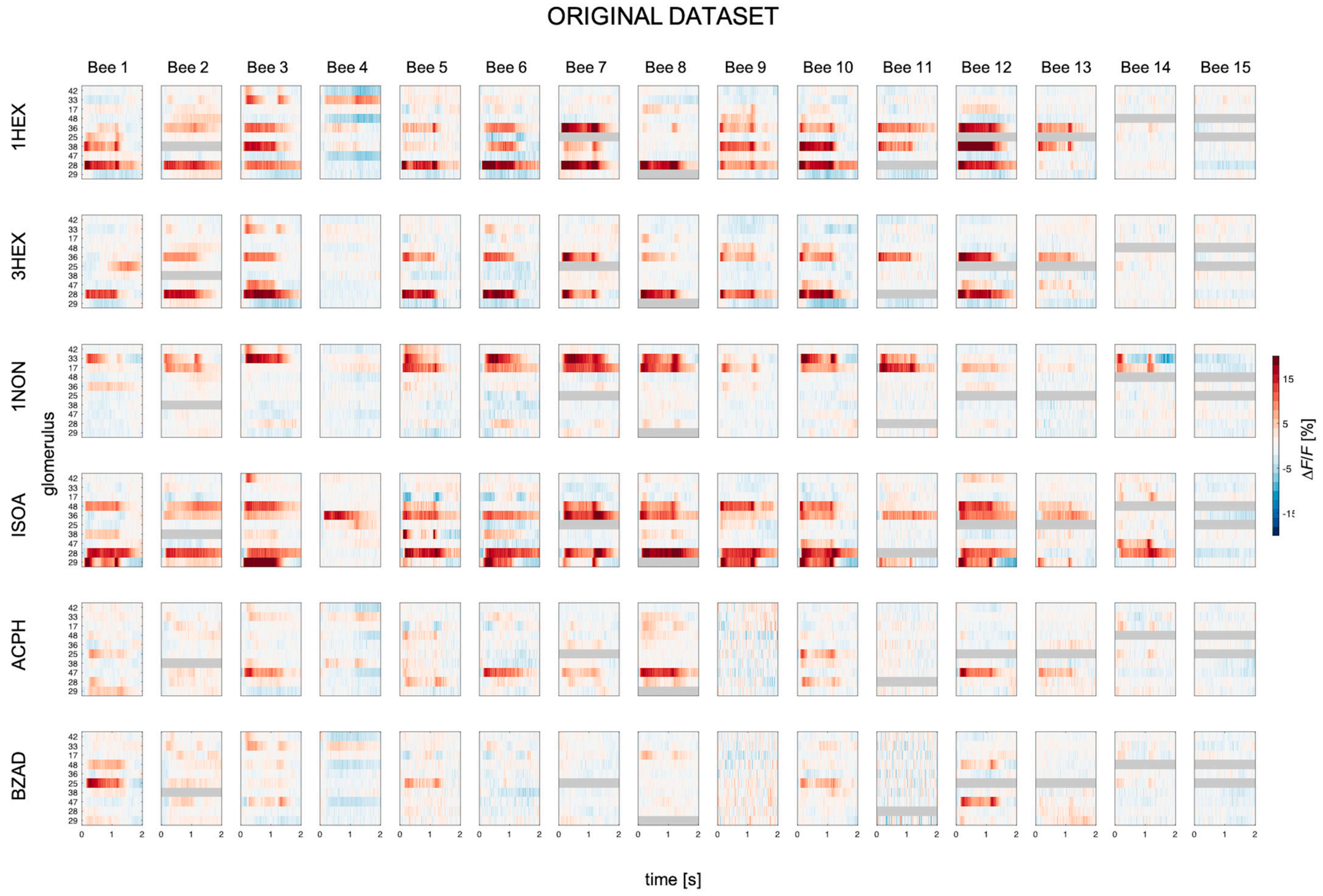

Supplementary Figure S2. Glomerular response profiles for 15 bees (columns) to 6 odorants (rows). Profiles are the mean responses of 30 stimulations. Stimulation interval from *t*=0 to 1s. Abbreviations: 1-hexanol, 1HEX; 3-hexanol, 3HEX; 1-nonanol, 1NON; isoamyl acetate, ISOA; acetophenone, ACPH; benzaldehyde, BZAD.

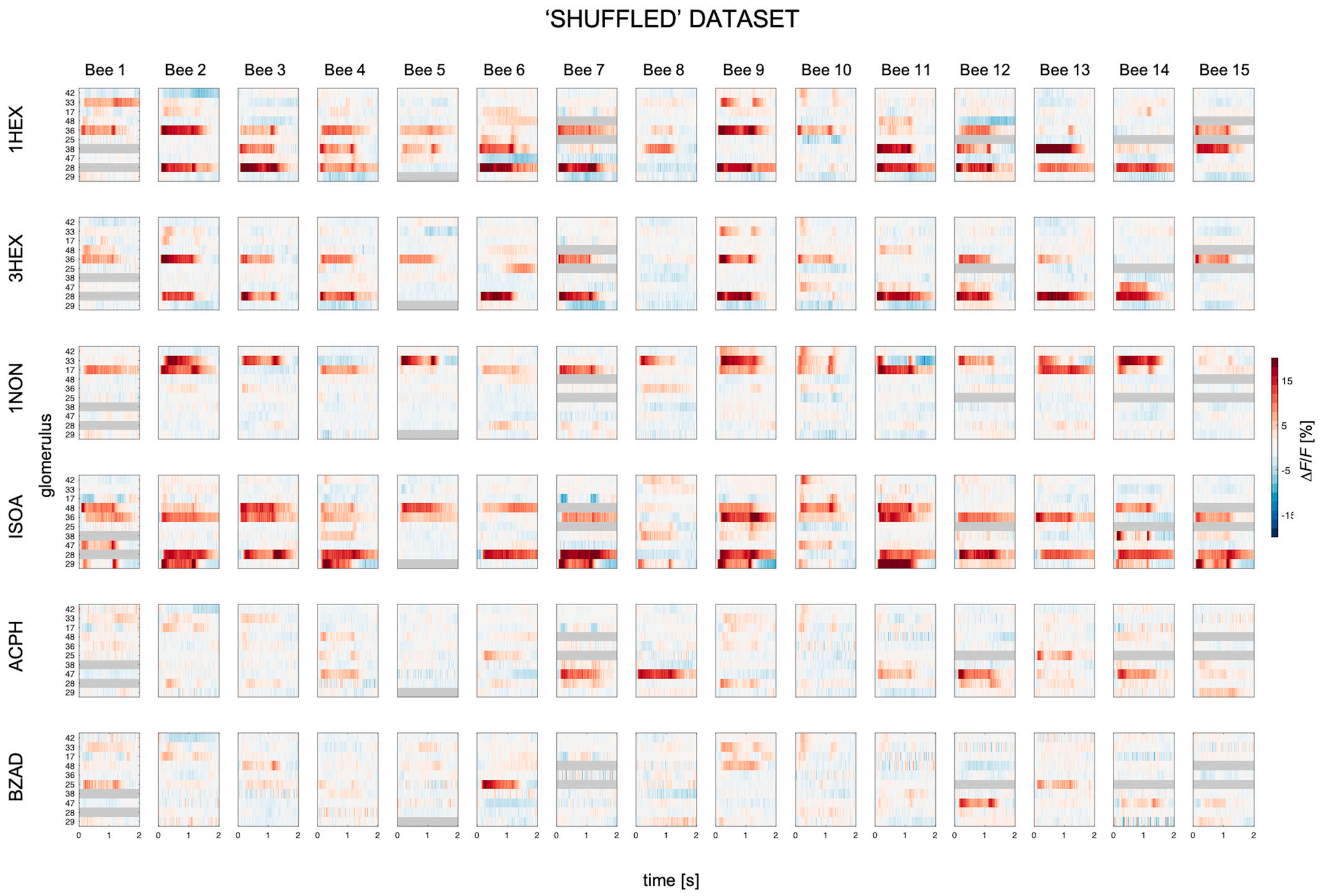

Supplementary Figure S3. Glomerular response profiles for 15 bees (columns) to 6 odorants (rows) after the shuffling procedure, i.e. each response map is composed by glomerular responses of the correct glomerulus, but extracted by different individuals. Profiles are the mean responses of 30 stimulations. Stimulation interval from t=0 to 1s. Abbreviations: 1-hexanol, 1HEX; 3-hexanol, 3HEX; 1-nonanol, 1NON; isoamyl acetate, ISOA; acetophenone, ACPH; benzaldehyde, BZAD.
